# Couinaud segment-aware deep learning on point clouds for major liver resection planning

**DOI:** 10.1007/s11548-026-03663-7

**Published:** 2026-05-08

**Authors:** Joy Rakshit, Janine Rothert, Georg Hille, Tobias Huber, Hauke Lang, Rabea Margies, Florentine Huettl, Sylvia Saalfeld

**Affiliations:** 1 Institute for Medical Informatics and Statistics, University Hospital Schleswig-Holstein, Kiel, Germany; 2 Department of General, Visceral and Transplant Surgery, University Medical Center of the Johannes Gutenberg University, Mainz, Germany

**Keywords:** Geometric deep learning, Couinaud segments, Couinaud-aware liver resection planning, Point cloud-based learning, Major hepatectomy planning using deep learning

## Abstract

**Purpose:**

In this study, we address the problem of automatic liver resection planning for major surgical procedures, including hemi-hepatectomy and extended hemi-hepatectomy, using deep learning. Motivated by clinical practice, where Couinaud liver segments are routinely used to describe tumor location and guide surgical decision-making, we investigate whether incorporating this anatomical information can improve model performance and clinical relevance.

**Methods:**

We propose a point cloud-based geometric deep learning approach based on a modified RandLA-Net architecture to predict liver resection zones. The model was trained and evaluated on 70 hemi-hepatectomy cases from Johannes Gutenberg University, Mainz, Germany (internal dataset). Two composite loss functions were evaluated: cross-entropy (CE) combined with intersection over union (IoU) and CE combined with Dice loss. For each loss function, models were trained with and without Couinaud segment information. Generalizability was assessed on an external dataset of 30 hemi-hepatectomy cases from the colorectal liver metastases (CRLM) cohort.

**Results:**

Both loss functions achieved comparable performance across the evaluated datasets, with CE + IoU consistently outperforming CE + Dice. On the internal test set, incorporating Couinaud segment information increased the IoU_mean_ from 0.787 to 0.804 and the F1-score from 0.864 to 0.870. A Wilcoxon signed-rank test on 15 paired cases confirmed a statistically significant improvement in IoU_mean_ (*p* = 0.030), with 80% of cases showing improvement. On the external dataset, IoU_mean_ improved from 0.666 to 0.702 and F1-score from 0.786 to 0.799 when Couinaud information was included. Excluding five anatomically complex cases, a Wilcoxon signed-rank test on the remaining 25 paired cases showed a significant improvement in IoU_mean_ (*p* = 0.019), with 68% of cases demonstrating improved performance.

**Conclusion:**

Explicit integration of Couinaud segment information improves both quantitative performance and clinical relevance in automatic major liver resection planning, particularly by better preserving critical vascular structures.

## Introduction

Liver resection is one of the primary therapeutic options for both primary and metastatic liver cancers. In primary liver cancer, the tumor originates within the liver itself, as observed in diseases such as hepatocellular carcinoma (HCC) and cholangiocarcinoma (bile duct cancer). In contrast, metastatic liver cancer arises when malignant cells spread to the liver from other primary sites, including the colon, breast, or lung, and so forth [2]. In both cases, patient-specific preoperative planning is crucial and must consider tumor extent, vascular anatomy, and preservation of sufficient functional liver tissue [1]. The importance of accurate liver resection planning is reflected in the global burden of the disease: In 2021, approximately 529,000 new liver cancer cases and 484,000 related deaths were reported worldwide, highlighting the need for precise and clinically informed surgical planning [14].

During preoperative planning, the Couinaud segmentation system is widely referred as the standard anatomical framework for describing tumor location and guiding surgical decision-making. Couinaud segmentation divides the human liver into eight functionally independent segments based on internal anatomical structures such as the portal vein, hepatic veins, and bile ducts [17]. Building on this anatomical concept, the Brisbane 2000 terminology classifies liver resections into three main categories: minor resections, combined or specific resections, and major resections [13]. Minor resections involve the removal of a single Couinaud segment, whereas combined or specific resections include two adjacent segments. Major resections comprise hemi-hepatectomy, involving the removal of either the right lobe (segments 5 to 8) or the left lobe (segments 2 to 4), as well as extended hemi-hepatectomy.

Furthermore, liver resection planning is an inherently complex and demanding task. The primary clinical objective is to achieve complete tumor removal with an adequate safety margin while preserving critical vascular structures and maintaining a sufficient future liver remnant (FLR) to ensure postoperative liver function. Beyond supporting surgical planning, an automatic resection prediction framework can also assist oncologists who do not directly perform surgery by enabling feasibility assessment prior to referral for operative treatment.

From a clinical perspective, tumor localization and resection strategies are routinely described in terms of Couinaud liver segments, reflecting regional variations in vascular perfusion and functional anatomy. This practice motivates the integration of Couinaud segment information into deep learning models to enable predictions that more closely align with clinical reasoning. Moreover, with the increasing adoption of virtual and augmented reality technologies for interactive surgical planning and training, geometric representations such as meshes and point clouds are particularly well suited for immersive visualization [5, 6, 16].

## Related work

Liver tumor resection requires precise preoperative planning due to complex vascular anatomy and the necessity of preserving adequate FLR. To support surgical decision-making, several visualization-based approaches have been proposed. Lu et al. [8] demonstrated that patient-specific 3D-printed liver models improve vascular-aware resection planning, while Huber et al. [5] and Huettl et al. [6] further enhanced planning accuracy by integrating 3D printing with virtual reality.

With the progress of medical image analysis, deep learning-based methods have increasingly been applied to automatic liver resection planning. Kang et al. [7] introduced a fully automatic framework using a liver-specific loss function to improve segmentation performance. Messaoudi et al. [9] proposed an attention-based network that leverages multiple anatomical segmentation masks and distance-maps to provide enhanced clinical context for FLR prediction.

More recently, geometric deep learning has been explored to model complex liver anatomy beyond traditional voxel-based representations. In previous work [11], we demonstrated that point cloud-based networks trained with composite loss functions can effectively predict liver resection volumes. Complementing this line of work, Chen et al. [3] proposed a semi-automatic planning system for middle hepatic vein–guided hemi-hepatectomy using 3D mesh-based resection pathways.

Anatomy-guided approaches that explicitly incorporate Couinaud segment information have also been investigated. Núñez et al. [10] showed that AI-assisted planning augmented with anatomical cues, such as lesion location relative to Couinaud segments, can reduce radiological workload without compromising accuracy. Similarly, Xie et al. [19] developed a cascaded 3D U-Net framework to segment liver anatomy, Couinaud segments, and hepatic tumors, enabling automatic major hepatectomy by resecting tumor-involved segments.

In contrast to existing methods, which either rely on voxel-based segmentation or use Couinaud information implicitly, our work explicitly integrates Couinaud segment information into a geometric deep learning framework for automatic major liver resection planning, aiming to improve both quantitative accuracy and clinical relevance.

## Materials and methods

### Data

**Fig. 1:**
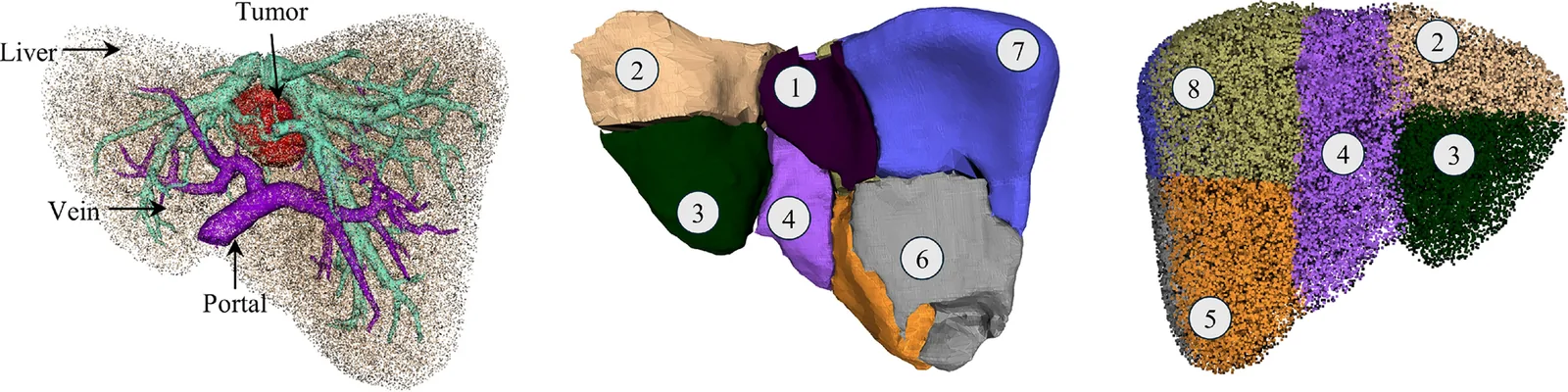
Example data showing the liver anatomy as a point cloud (left image) and the corresponding Couinaud segments represented in mesh (middle-image) and point cloud (right-image) formats

The methods were trained and evaluated on an internal dataset and further validated on an external dataset. For both datasets, medical image data, mesh data and point cloud data were analyzed. The internal dataset comprises 70 (extended) hemi-hepatectomy cases from the University Medical Center of Johannes Gutenberg University Mainz, Germany, including intrahepatic cholangiocarcinoma (62), hepatocellular carcinoma (4), and colorectal liver metastases (4), with 39 right and 31 left hemi-hepatectomies. The internal dataset comprises 70 hemi-hepatectomy cases acquired from University Medical Center of the Johannes Gutenberg University Mainz, Germany. Experienced clinicians manually segmented the raw computed tomography (CT) volumes using Fuji Synapse to delineate vascular structures, tumors, resection volumes, and the future liver remnant. These segmentations were subsequently converted into mesh representations. These mesh models were subsequently transformed into point cloud format by adapting the point cloud preparation pipeline proposed by Rakshit et al. [11]. The left image in Fig. 1 illustrates an example of the point cloud representation of anatomical structures used for network training.

The external dataset comprises 30 (extended) hemi-hepatectomy cases from the colorectal liver metastases (CRLM) cohort, used for further validation [12]. The dataset includes contrast-enhanced preoperative CT scans from a single-institution cohort of patients with pathologically confirmed colorectal liver metastases who underwent hepatic resection. Cases were selected in which hemi-hepatectomy was performed and that provided sufficient anatomical coverage, requiring each CT volume to contain at least 75 slices for reliable reconstruction of major vascular structures.

In addition, patient-specific Couinaud liver segmentations were generated using the open-source framework *TotalSegmentator* from CT volumes. [18]. TotalSegmentator incorporates the global and local context U-Net (GLC-U-Net) architecture proposed by Tian et al. [15], which jointly captures intra-slice features and three-dimensional contextual information through an attention-based mechanism. The model was trained and evaluated on 43,205 annotated CT slices, achieving Dice scores of 98.51% for liver segmentation and 92.46% for Couinaud segment segmentation. The middle and right images in Fig. 1 illustrate the resulting mesh and point cloud representations, respectively, of the Couinaud liver segments (segments 1 to 8) for the same example case. The previously generated liver point cloud was accurately associated with the Couinaud segmentations using a GPU-accelerated signed distance function (SDF)-based registration strategy. This information is subsequently utilized during model training. Specifically, each point in the point cloud is assigned a discrete label ranging from 1 to 8, corresponding to its anatomical location within the Couinaud liver segmentation. This per-point segment identifier is incorporated as an additional feature to provide region-specific anatomical context to the network. On average, the complete point cloud preparation process for a single case requires approximately 40 s.

### Model training

Prior work by Rakshit et al. [11] demonstrated that RandLA-Net, proposed by Hu et al. [4], achieves strong performance in liver segmentation when trained using a linear combination of cross-entropy (CE) and intersection over union (IoU) loss. Motivated by these findings, we adopt RandLA-Net as the baseline architecture in this study and evaluate two composite loss formulations: a linear combination of CE and IoU, and a linear combination of CE and Dice (F1) loss. Furthermore, the network is extended to incorporate additional anatomical information in the form of *Couinaud segment* features. To assess the contribution of this auxiliary information, experiments are conducted with and without Couinaud features, evaluation of their impact on segmentation performance across both internal and external datasets.

We employed a grid search strategy to determine the optimal hyperparameter settings. The resulting configuration, Table 1, was largely consistent with that identified in our previous experiments, with the exception of the number of input points, which was adjusted according to the specific dataset used. Experiments were performed on a workstation with an Intel Xeon E5-2630 v4 CPU (2.20 GHz) and an NVIDIA GeForce GPU (11 GB VRAM), using TensorFlow 1.15 with CUDA 10.2 and cuDNN 7.

**Table 1:** Training parameters

Parameter	Value
k-Nearest Neighbor Information/Point	16
Training Batch Size	4
Training Steps	500
Max Epoch	100
Learning Rate	1.00e-02
Number of Input Points	10 × 10^4^
Number of Layers	5
Feature Dimensions	16, 64, 128, 256, 512
Sampling Ratio	4, 4, 4, 4, 2
Augment Scale Anisotropic	1
Augment Symmetry	1, 0, 0
Augment Rotation Vertical	0.5
Augment Scale	Min, Max: 0.8, 1.2
Augment Noise (Gaussian)	0.001

During training, the (internal) dataset of 70 cases was divided into 50 training, 5 validation, and 15 testing cases. Prior to random assignment, cases were stratified primarily by resection type (right vs. left hemi-hepatectomy) and secondarily by tumor location with respect to Couinaud segments. This ensured a balanced representation of clinically relevant anatomical variations across all splits.

**Fig. 2:**
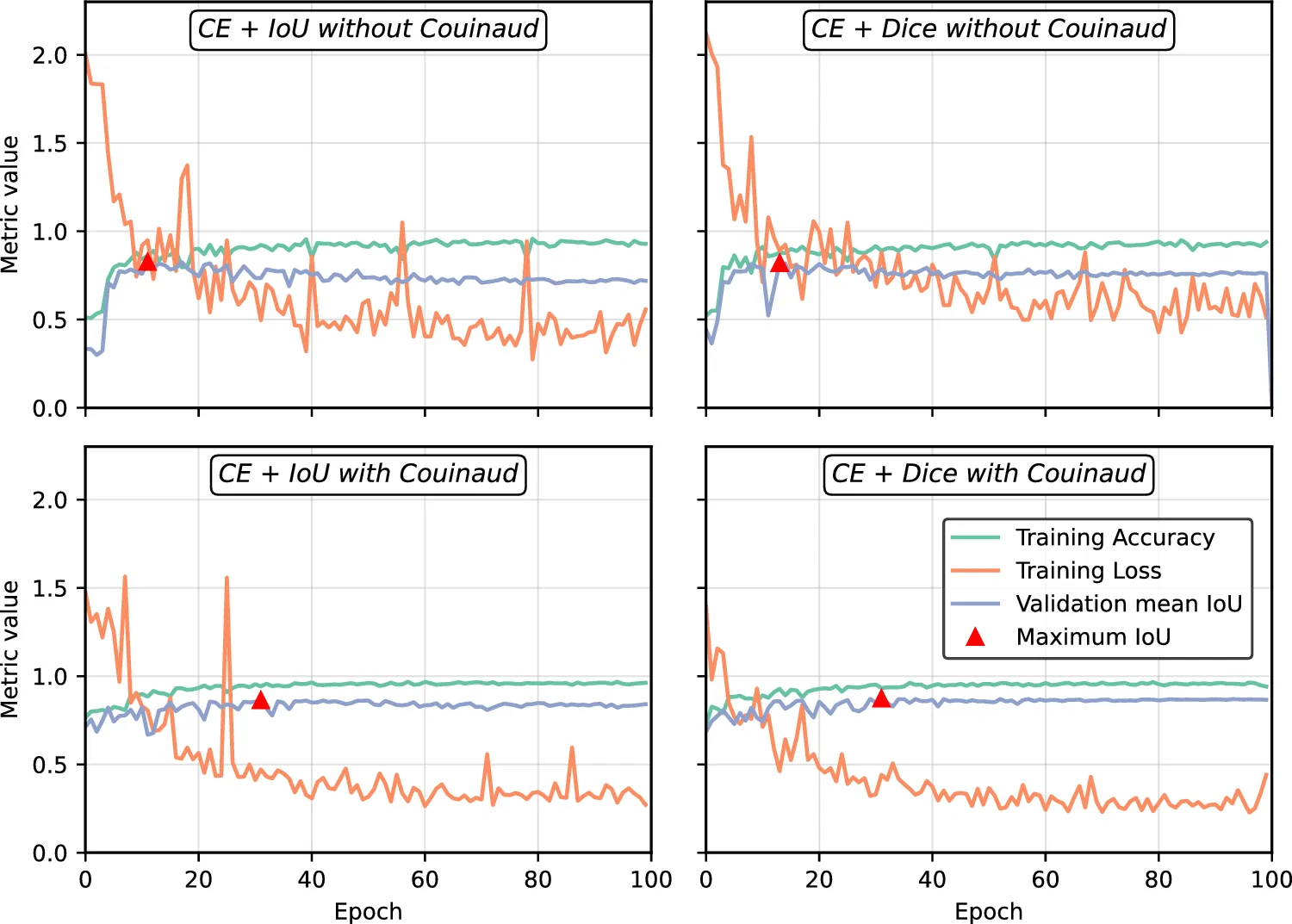
Training curves for models trained without (top-row) and with (bottom-row) Couinaud information, using CE + IoU (left) and CE + Dice (right) loss functions

Figure 2 presents the training and validation performance curves. In the absence of Couinaud information, the CE + IoU and CE + Dice loss formulations achieve mean validation IoU (marked as red triangles) scores of 82.61% and 81.99%, respectively. When Couinaud information is incorporated, these scores improve substantially to 87.35% and 86.32%.

## Results

### Mainz dataset

Table 2 summarizes the quantitative results on the internal (Mainz) test dataset. Overall, both loss function configurations demonstrate comparable performance. Nevertheless, models incorporating Couinaud information demonstrate improved overall performance, as reflected by higher IoU_mean_, F1-scores and FLR compared to their counterparts trained without anatomical segment guidance. To further assess the robustness of the results across different experimental settings, we report standard deviation (SD) along with the mean values of these metrics, thereby capturing variability across cases.

**Table 2:** Test results on internal (Mainz) dataset

Experiment	Accuracy	Precision	Recall	F1_Score	IoU_mean_	FLR (%)
CE + IoU	0.883	0.914	**0.833**	0.864 ± 0.111	0.787 ± 0.153	55.78 ± 18.91
CE + IoU_Couinaud_	**0.893**	**0.956**	0.815	**0.870 ± 0.117**	**0.804 ± 0.151**	**58.90 ± 17.97**
CE + Dice	0.862	0.910	**0.810**	0.844 ± 0.113	0.745 ± 0.160	54.73 ± 22.70
CE + Dice_Couinaud_	**0.889**	**0.968**	0.786	**0.857 ± 0.113**	**0.790 ± 0.140**	**59.77 ± 19.59**

Bold values highlight the best-performing results for each loss function under both Couinaud-aware and non-Couinaud configurations

*Note: Ground truth FLR (mean ± SD) across the test set: 51.42 ± 18.02

For the best-performing CE + IoU model configuration, 12 of the 15 test cases (80%) showed an improvement in IoU_mean_ when Couinaud segment information was included. Among the improved cases, the median increase in IoU_mean_ was 0.031 (range 0.003-0.112), while the remaining cases showed a marginal decrease with a median change of -0.004. Furthermore, a Wilcoxon signed-rank test conducted on the 15 paired IoU_mean_ values indicated a statistically significant improvement (*p* = 0.030).

**Fig. 3:**
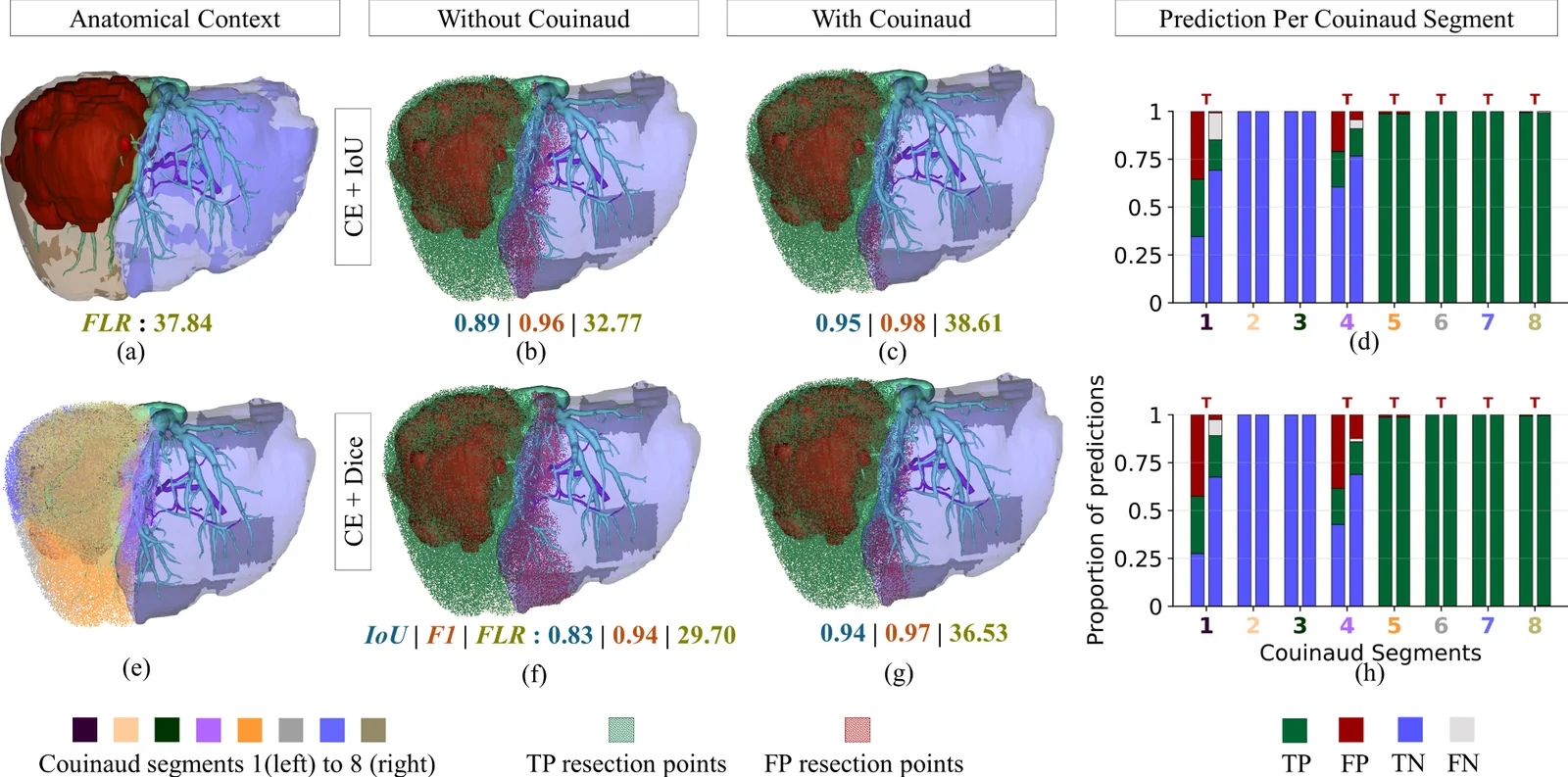
An exemplary (or qualitative) test case from internal data. (**a**) Tumor (dark red opaque) location in resection volume (brown transparent) and future liver remnant (blue transparent); (**e**) Couinaud segment point cloud overlay on resection volume. CE + IoU (**b**–**c**) and CE + Dice (**f**–**g**) show predictions without and with Couinaud information. Couinaud segment-wise prediction proportions are shown in pair bars (**d**, **h**; left = without, right = with). Dark red ‘T’ marks tumor presence in the corresponding segments denoting x-axis

Figure 3 illustrates a representative right hemi-hepatectomy test case from the internal dataset. Qualitatively, incorporating Couinaud segment information substantially reduces false-positive (FP) predictions as red points, while true-positive (TP) predictions are shown in green, thereby preserving the middle hepatic vein and resulting in more clinically relevant resection predictions. The IoU_mean_, F1-scores and FLR in percentage for each segmentation model are reported below the images (blue: IoU_mean_, orange: F1-score and olive-green: FLR). For this case, the CE + IoU model achieves the highest performance, with a IoU_mean_ of 0.95, an F1-score of 0.98 and an FLR of 38.61.

### Colorectal liver metastases dataset

Table 3 presents the qualitative test results on the external dataset, colorectal liver metastases (CRLM) [12]. It is evident that the model trained with Couinaud information outperformed the counterpart trained without Couinaud information. Additionally, the model trained with the CE + IoU loss function achieved slightly better performance than the CE + Dice loss function.

**Table 3:** Test results on external (CRLM) dataset

Experiment	Accuracy	Precision	Recall	F1_Score	IoU_mean_	FLR (%)
CE + IoU	0.804	**0.911**	0.729	0.786 ± 0.176	0.666 ± 0.174	**53.57 ± 20.38**
CE + IoU_Couinaud_	**0.826**	0.908	**0.755**	**0.799 ± 0.205**	**0.702 ± 0.192**	51.82 ± 20.51
CE + Dice	0.785	**0.934**	0.672	0.750 ± 0.195	0.636 ± 0.171	**57.27 ± 22.34**
CE + Dice_Couinaud_	**0.822**	0.913	**0.740**	**0.790 ± 0.203**	**0.690 ± 0.181**	52.03 ± 21.61

Bold values highlight the best-performing results for each loss function under both Couinaud-aware and non-Couinaud configurations

*Note: Ground truth FLR (mean ± SD) across the test set: 42.55 ± 17.59

Performance variations were noted in 5 out of 30 CRLM cases, particularly for tumors located in anatomically complex regions that were underrepresented in the training dataset (detail in Sect. "Discussion"). For the five challenging cases, the distribution of IoU_mean_ values had a median of 0.459 (without Couinaud) and 0.360 (with Couinaud). In contrast, the distribution for the remaining cases showed higher median IoU_mean_ values of 0.714 (without Couinaud) and 0.766 (with Couinaud). Excluding these cases, a Wilcoxon signed-rank test conducted on the remaining 25 paired test cases revealed a statistically significant improvement in IoU_mean_ when Couinaud segment information was included (*p* = 0.019). Notably, 68% of the cases exhibited improved performance, with a median IoU_mean_ increase of 0.085 (maximum: 0.330 and minimum: 0.051).

**Fig. 4:**
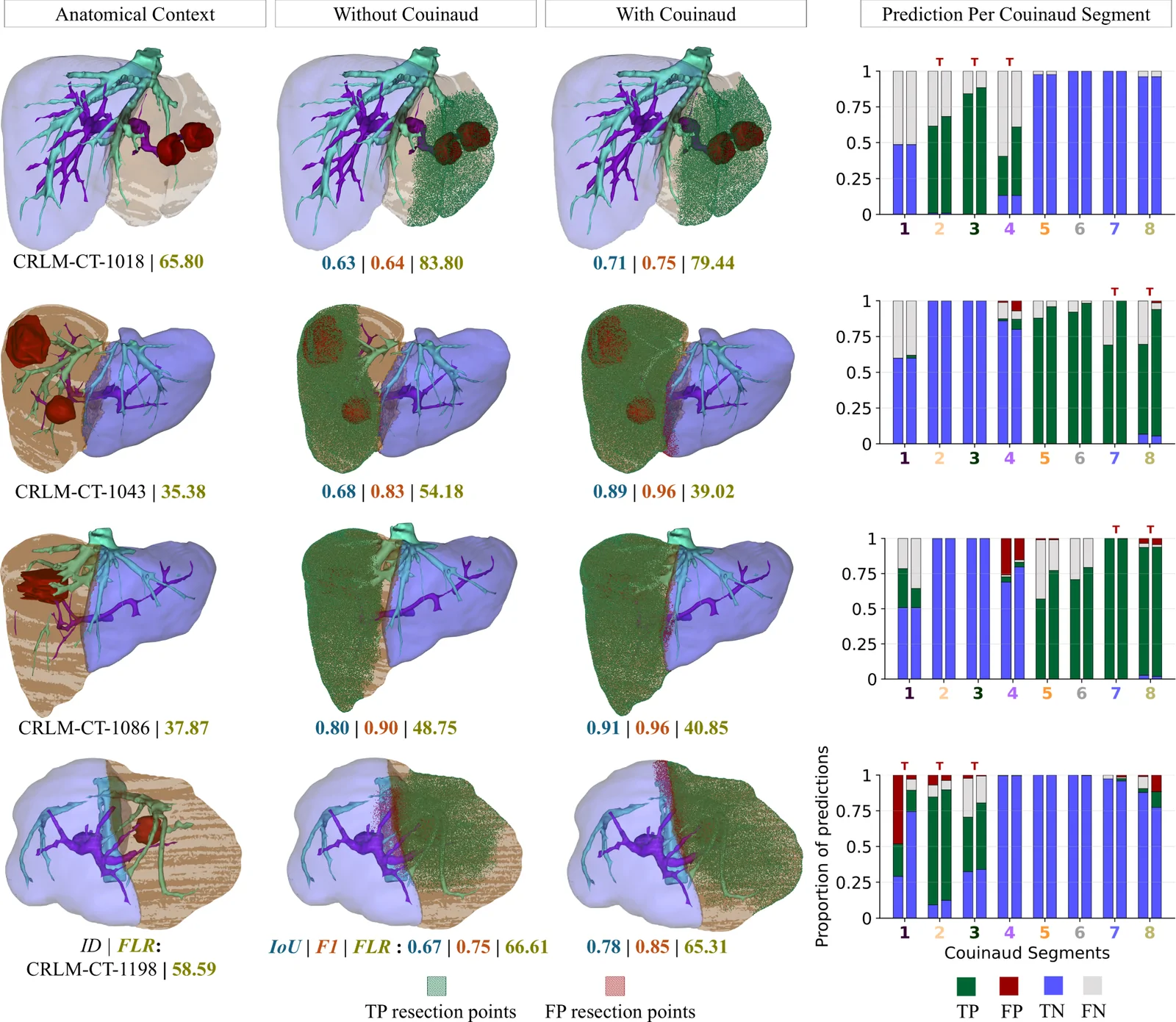
Qualitative results on CRLM dataset using the CE + IoU loss. The *left column* shows the anatomical context, including the tumor (opaque-red), resection volume (transparent-brown), and future liver remnant (transparent-blue). The *middle columns* present segmentation results from models trained without and with Couinaud segment information. The *right column* illustrates Couinaud segment-wise prediction proportions as paired bar plot (left = without, right = with Couinaud information)

Figure 4 illustrates the qualitative evaluation on the CRLM dataset. The case identifiers shown below each example provided in anatomical context column. The next two columns present segmentation results obtained using the CE + IoU loss function for models trained without and with Couinaud segment information, respectively, together with the corresponding IoU and F1-scores for each case.

In terms of computational efficiency, the proposed model requires approximately 12 s per patient (case) for inference.

The Couinaud-aware model demonstrates a clear reduction in FP predictions (red points in transparent-brown region), particularly in regions near vascular structures. Additionally, a reduction in FN errors (unoccupied space in transparent-brown region), resulting in resection predictions that more closely align with clinically acceptable hemi-hepatectomy boundaries. Consistently, the paired bar plots indicate higher proportions of TP (green) and TN (blue) predictions for the Couinaud-aware model, reflecting improved segmentation quality.

## Discussion

From a learning perspective, the Couinaud-aware model demonstrates faster convergence compared to its non-Couinaud counterpart. As shown in Fig. 2 (in Sect. "Model Training"), training with Couinaud information exhibits a faster convergence behavior, as evidenced by higher validation performance from the first evaluation epoch for both loss functions. This indicates that the inclusion of Couinaud segment features facilitates more effective learning compared to the corresponding models trained without Couinaud-context. Furthermore, Couinaud-aware models reduce both false negatives and false positives as shown in Figs. 3 and 4*,* and better preserve critical vascular structures, leading to predictions that are more consistent with clinical practice and enhancing their potential clinical applicability. This can be further explained by the fact that Couinaud segments inherently reflect the branching structure of the portal venous system and thus encode functionally and surgically relevant anatomical information. In this sense, they provide an implicit surrogate for vascular structures, which may guide the model toward more anatomically consistent and clinically meaningful resection predictions.

**Fig. 5:**
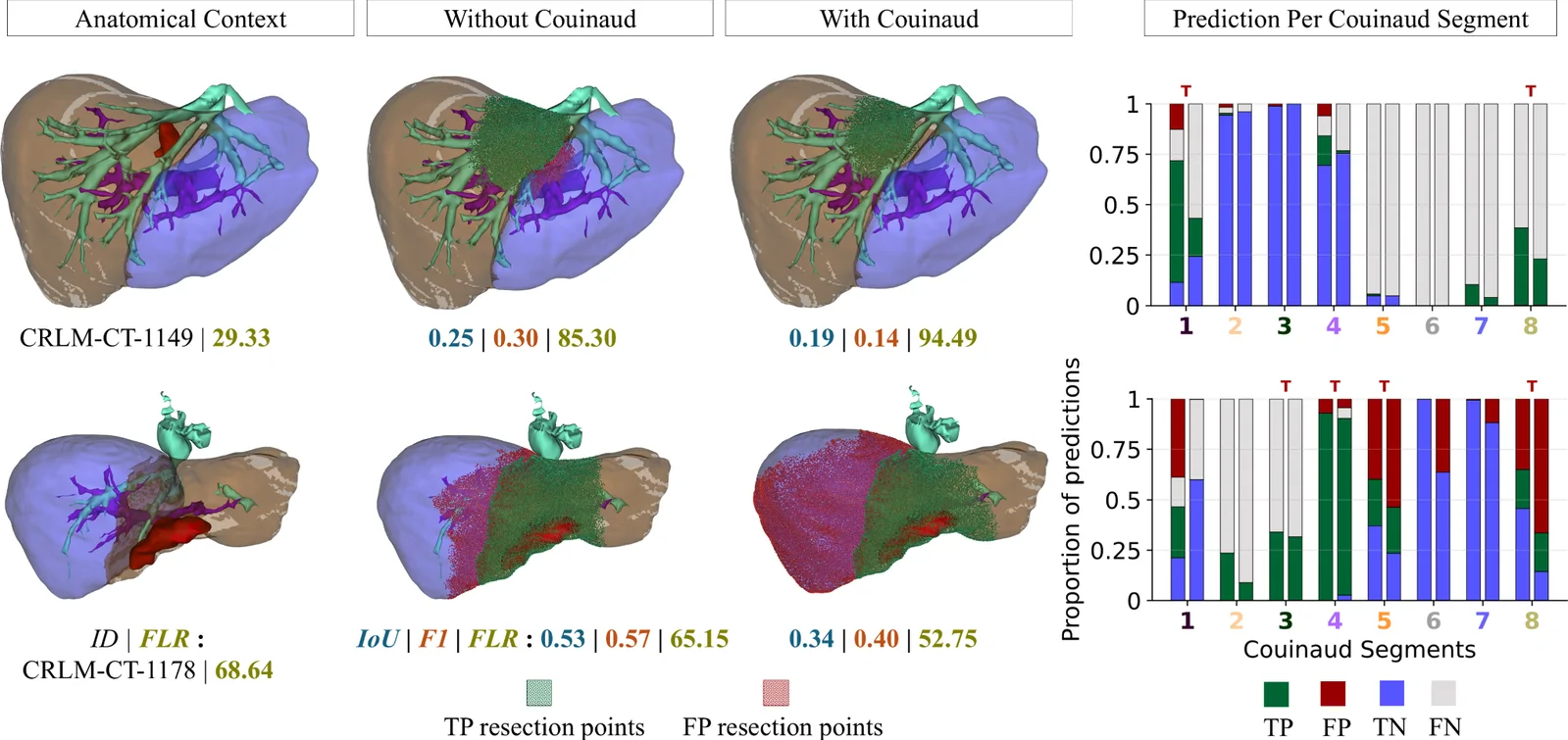
Example of challenging anatomical test cases (from external data, CRLM)

It is observed that models, with and without Couinaud information both, show reduced performance for cases with tumors located near the central region of the liver or if the tumors are particularly close to the liver surface (e.g., the five outlier cases of the external dataset, CRLM). Such anatomically complex scenarios were underrepresented in the selected cases of the training dataset. For these 5 cases, the median IoU_mean_ was 0.459 without Couinaud and 0.360 with, compared to 0.714 and 0.766 for the remaining cases. For example, a tumor in segments 1 and 8 (Fig. 5, first row) is predicted similarly by both models, but the Couinaud-aware model better preserves the middle hepatic vein. Another tumor spanning segments 3, 4, 5, and 8 near the liver boundary (Fig. 5, second row) leads the model to overpredict right-lobe segments, while the ground truth corresponds to a left extended hemi-hepatectomy.

While the definition of Couinaud segments is standardized, the precise delineation of segment boundaries may vary slightly across clinicians. In this study, patient-specific Couinaud segmentations were generated using a well-established automated framework, which already led to measurable improvements in prediction performance. Nevertheless, additional gains may be achievable by employing Couinaud segmentations derived from the same clinical experts who define the surgical resection.

Notably, in rare extreme cases, particularly when the tumor occupies a substantial portion of the liver, TotalSegmentator may fail to accurately delineate liver parenchyma due to the inclusion of tumor tissue, leading to unreliable segment predictions. These cases (2 out of all cases in the internal dataset) were therefore excluded from the analysis.

The model tends to preserve a larger portion of liver parenchyma compared to the ground truth, as reflected by consistently higher FLR values across experiments, while still ensuring adequate tumor coverage with clinically acceptable safety margins. Notably, the Couinaud-aware configurations demonstrate a stronger tendency to preserve parenchyma compared to their non-Couinaud counterparts, suggesting that the incorporation of anatomical context supports more conservative and potentially function-preserving resection strategies. This behavior indicates that the proposed approach may offer flexibility in balancing oncological safety and parenchymal preservation, which is an important consideration in clinical practice. Moreover, resection planning strategies can differ across clinical centers depending on available infrastructure and surgeon experience. In such scenarios, fine-tuning the proposed model on a small institution-specific dataset could enable it to adapt to local surgical practices and improve its clinical applicability.

Due to the absence of directly comparable studies focusing on automatic hemi-hepatectomy planning, a direct quantitative comparison with prior work is not feasible. In future, broader validation across larger and more diverse multicenter datasets would be valuable. Nevertheless, relative performance can be contextualized with existing methods addressing general liver resection. Messaoudi et al. [9] reported an image-based deep learning approach for general liver resection, achieving an F1-score of 0.89, an IoU of 0.79, and a precision of 0.95. Similarly, Rakshit et al. [11] proposed a point cloud-based method for general liver resection, reporting an F1-score of 0.84, an IoU of 0.76, and a precision of 0.86. In comparison, our method targets the more specific clinical task of hemi-hepatectomy prediction. On the internal test dataset, our Couinaud-aware model achieves an F1-score of 0.87, an IoU of 0.80, and a precision of 0.96, while on the external dataset it attains an F1-score of 0.80, an IoU of 0.70, and a precision of 0.91. These results demonstrate competitive performance and highlight the potential of geometric deep learning for future research in liver resection planning.

## Conclusion

In this work, we investigated automatic liver resection planning with a particular focus on major resections, including (extended) hemi-hepatectomy. Our results demonstrate that incorporating anatomical context in the form of patient-specific Couinaud segment information consistently improves model performance compared to a baseline approach without such information. By leveraging a geometric deep learning formulation on point cloud representations, the proposed Couinaud-aware model produces predictions that are more clinically meaningful, particularly by better preserving critical vascular structures and maintaining an adequate future liver remnant (FLR), thereby aligning more closely with surgical decision-making. In addition, the proposed pipeline demonstrates practical computational efficiency, requiring approximately 40 s per patient for point cloud generation and Couinaud segments’ (achieved through TotalSegmentator) assignment, followed by around 12 s for resection zone prediction.

The proposed method was trained and evaluated on a clinical dataset from internal dataset and further validated on the publicly available colorectal liver metastases (CRLM) dataset [12], to demonstrate robustness across datasets. Two composite loss functions, CE combined with IoU and CE combined with Dice were evaluated, with CE + IoU consistently yielding slightly better results.

The best-performing configuration, trained with Couinaud segment information and the CE + IoU loss, achieved a IoU_mean_ of 0.804 and an F1-score of 0.870 on the internal test set, compared to 0.787 and 0.864, respectively, for the corresponding model trained without Couinaud information. Similarly, on the external dataset, the inclusion of Couinaud segments improved the IoU_mean_ from 0.666 to 0.702 and the F1-score from 0.786 to 0.799. Furthermore, Wilcoxon signed-rank tests performed on paired IoU_mean_ values from the test datasets (internal: *p* = 0.030; external after excluding outlier cases: *p* = 0.019) provide statistical support for the performance gains achieved by incorporating Couinaud segment information.

In future work, we plan to extend this concept by explicitly incorporating vascular tree information, thereby moving from indirect anatomical surrogates to direct modeling of clinically critical structures. Here, clinically oriented metrics such as vessel proximity could be included and the method could be integrated into interactive planning environments (e.g., AR/VR-based systems), which would allow surgeons to directly assess and adapt the predicted resection regions in a clinically meaningful workflow.

Overall, these findings indicate that integrating Couinaud segment information provides valuable anatomical context, enabling the network to enhance segmentation accuracy and produce more clinically relevant liver resection predictions.

## Data Availability

The code is publicly available under https://github.com/AIinMedicalApplications/LIZARD-CouinaudAwareResection.

## References

[1] Alirr OI, Rahni AAA (2020) Survey on liver tumour resection planning system: steps, techniques, and parameters. J Digit Imag 33(2):304–323. 10.1007/s10278-019-00262-8 PMC7165210 31428898

[2] Ananthakrishnan A, Gogineni V, Saeian K (2006) Epidemiology of primary and secondary liver cancers. Semin Intervent Radiol 23(1):47–63. 10.1055/s-2006-939841 PMC3036307 21326720

[3] Chen, W. et al. (2013) Planning of middle hepatic vein-guided hemihepatectomy: resection pathway construction and optimization, 8090, S 55–64. 10.1007/978-3-642-40843-4_7

[4] Hu Q, Yang B, Xie L, Rosa S, Guo Y, Wang Z, Trigoni N, Markham A (2019) RandLA-net: efficient semantic segmentation of large-scale point clouds. 10.48550/arXiv.1911.11236. https://arxiv.org/pdf/1911.11236 34033533

[5] Huber T, Hanke LI, Boedecker C, Vradelis L, Baumgart J, Heinrich S, Bartsch F, Mittler J, Schulze A, Hansen C, Hüttl F, Lang H (2022) Patient-individualized resection planning in liver surgery using 3D print and virtual reality (i-LiVR)-a study protocol for a prospective randomized controlled trial. Trials 23(1):403. 10.1186/s13063-022-06347-0 PMC9100295 35562806

[6] Huettl F, Saalfeld P, Hansen C, Preim B, Poplawski A, Kneist W, Lang H, Huber T (2021) Virtual reality and 3D printing improve preoperative visualization of 3D liver reconstructions-results from a preclinical comparison of presentation modalities and user’s preference. Ann Transl Med 9(13):1074. 10.21037/atm-21-512 PMC8339861 34422986

[7] Kang CM, Ku HJ, Moon HH, Kim S-E, Jo JH, Choi YI, Shin DH (2024) Predicting safe liver resection volume for major hepatectomy using artificial intelligence. J Clin Med 13(2):381. 10.3390/jcm13020381 PMC10816299 38256518

[8] Lu Y, Chen X, Han F, Zhao Q, Xie T, Wu J, Zhang Y (2023) 3D printing of self-healing personalized liver models for surgical training and preoperative planning. Nat Commun 14(1):8447. 10.1038/s41467-023-44324-6 PMC10730511 38114507

[9] Messaoudi H, Abbas M, Badic B, Ben Salem D, Belaid A, Conze P-H (2025) Automatic future remnant segmentation in liver resection planning. Int J Comput Assist Radiol Surg 20(5):837–845. 10.1007/s11548-025-03331-2 39961898

[10] Núñez L, Ferreira C, Mojtahed A, Lamb H, Cappio S, Husainy MA, Dennis A, Pansini M (2024) Assessing the performance of AI-assisted technicians in liver segmentation, Couinaud division, and lesion detection: a pilot study. Abdom Radiol (NY) 49(12):4264–4272. 10.1007/s00261-024-04507-1 PMC11522103 39123052

[11] Rakshit J, Kreher R, Huber T, Lang H, Huettl F, Saalfeld S (2025) Geometric deep learning adapted to prediction of liver resection zone. Comput Biol Med 197(Pt B):111103. 10.1016/j.compbiomed.2025.111103 41005230

[12] Simpson AL, Peoples J, Creasy JM, Fichtinger G, Gangai N, Lasso A, Keshava Murthy KN, Shia J, D’Angelica MI, Do RKG (2023) Preoperative CT and survival data for patients undergoing resection of colorectal liver metastases (Colorectal-Liver-Metastases). Cancer Imaging Arch. 10.7937/QXK2-QG03 PMC10847495 38321027

[13] Strasberg SM, Belghiti J, Clavien P-A, Gadzijev E, Garden JO, Lau W-Y, Makuuchi M, Strong RW (2000) The Brisbane 2000 terminology of liver anatomy and resections. HPB 2(3):333–339. 10.1016/S1365-182X(17)30755-4

[14] Tan EY, Danpanichkul P, Yong JN, Yu Z, Tan DJH, Lim WH, Koh B, Lim RYZ, Tham EKJ, Mitra K, Morishita A, Hsu Y-C, Yang JD, Takahashi H, Zheng M-H, Nakajima A, Ng CH, Wijarnpreecha K, Muthiah MD, Singal AG, Huang DQ (2025) Liver cancer in 2021: global burden of disease study. J Hepatol 82(5):851–860. 10.1016/j.jhep.2024.10.031 39481652

[15] Tian J, Liu L, Shi Z, Xu F (2019) automatic couinaud segmentation from CT volumes on liver using GLC-UNet. In: Suk H-I, Liu M, Yan P, Lian C (Hrsg) Machine Learning in Medical Imaging, Bd 11861. Springer International Publishing, Cham, S 274–282

[16] Turchet L, Gentilini F, Malandra S, Veccia A, Antonelli A, Scoffone C (2025) Medical training in virtual reality: A gamification approach. Virtual Real. 10.1007/s10055-025-01159-4

[17] Wang M, Jin R, Lu J, Song E, Ma G (2022) Automatic CT liver Couinaud segmentation based on key bifurcation detection with attentive residual hourglass-based cascaded network. Comput Biol Med 144:105363. 10.1016/j.compbiomed.2022.105363 35290810

[18] Wasserthal J, Breit H-C, Meyer MT, Pradella M, Hinck D, Sauter AW, Heye T, Boll DT, Cyriac J, Yang S, Bach M, Segeroth M (2023) TotalSegmentator: robust segmentation of 104 anatomic structures in CT images. Radiol Artif Intell 5(5):e230024. 10.1148/ryai.230024 PMC10546353 37795137

[19] Xie T, Li Y, Lin Z, Liu X, Zhang X, Zhang Y, Zhang D, Cheng G, Wang X (2023) Deep learning for fully automated segmentation and volumetry of Couinaud liver segments and future liver remnants shown with CT before major hepatectomy: a validation study of a predictive model. Quant Imag Med Surg 13(5):3088–3103. 10.21037/qims-22-1008 PMC10167444 37179921

